# Aqueous Sol-Gel Synthesis of Different Iron Ferrites: From 3D to 2D

**DOI:** 10.3390/ma14061554

**Published:** 2021-03-22

**Authors:** Justinas Januskevicius, Zivile Stankeviciute, Dalis Baltrunas, Kęstutis Mažeika, Aldona Beganskiene, Aivaras Kareiva

**Affiliations:** 1 Institute of Chemistry, Vilnius University, Naugarduko st. 24, LT-03225 Vilnius, Lithuania; justinas.januskevicius@chgf.vu.lt (J.J.); zivile.stankeviciute@chf.vu.lt (Z.S.); aldona.beganskiene@chf.vu.lt (A.B.); 2 Center of Physical Sciences and Technology, LT-02300 Vilnius, Lithuania; dalis.baltrunas@ftmc.lt (D.B.); kestutis.mazeika@ftmc.lt (K.M.)

**Keywords:** sol-gel synthesis, iron ferrites, garnet, perovskites, thin films

## Abstract

In this study, an aqueous sol-gel synthesis method and subsequent dip-coating technique were applied for the preparation of yttrium iron garnet (YIG), yttrium iron perovskite (YIP), and terbium iron perovskite (TIP) bulk and thin films. The monophasic highly crystalline different iron ferrite powders have been synthesized using this simple aqueous sol-gel process displaying the suitability of the method. In the next step, the same sol-gel solution was used for the fabrication of coatings on monocrystalline silicon (100) using a dip-coating procedure. This resulted, likely due to substrate surface influence, in all coatings having mixed phases of both garnet and perovskite. Thermogravimetric (TG) analysis of the precursor gels was carried out. All the samples were investigated by X-ray powder diffraction (XRD) analysis. The coatings were also investigated by scanning electron microscopy (SEM), atomic force microscopy (AFM) and Mössbauer spectroscopy. Magnetic measurements were also carried out.

## 1. Introduction

The interest in perovskite and garnet ceramics has been growing steadily over recent years. This is because these materials are promising in a variety of fields, such as solar cells [1,2], magneto-optical materials [3,4], gas sensors, superconductors [5], new memory devices [6], and many others [7,8,9,10,11,12,13,14]. The garnet structure has a general formula C_3_A_2_D_3_O_12_, where the first three are cations in dodecahedral, octahedral, and tetrahedral positions, respectively [15,16]. The structure is relatively rigid, with low space-filling, and as such, can accept a wide range of substitutes [7,15]. Perovskite, which has the formula ABX_3_, is also known for being capable of accepting a wide range of substitutes. The reason is very different, however; where the garnet structure is rigid, the perovskite structure is flexible, prone to deformation, which disrupts symmetry [16,17]. This disruption of symmetry can sometimes be very advantageous; for example, it can be the cause of ferroelectricity and similar phenomena. As such, some tentative control over the properties from a structural design standpoint is possible, if the ionic radii and electronic structures of constituent ions are considered [18,19,20].

The materials investigated in this particular work, namely, yttrium iron garnet (YIG), yttrium iron perovskite (YIP), and terbium iron perovskite (TIP) are interesting mainly due to their magnetic properties. YIG, which has the formula Y_3_Fe_5_O_12_, has iron occupying both the octahedral and tetrahedral positions. One consequence of this is that the material, due to uncompensated spins, displays ferrimagnetic behavior, among other interesting phenomena. As such, it is a material that has already found some use (due to Faraday rotation) even in bulk form, with easily producible, high-quality coatings being a desirable development [21]. YIP, conversely, is a material that has not yet attracted much interest but has been synthesized and investigated as a potential multiferroic, reportedly displaying ferroelectric and antiferromagnetic properties [22,23]. TIP is in a similar situation as YIP, only the lattice is slightly larger. It is, nonetheless, a material that has shown interesting properties of its own and is worth investigating in its own right [24,25,26], as well as for comparison purposes to the other materials mentioned.

Considering the wide range of applications of coatings, it is necessary to search for inexpensive, simple and upscalable synthetic approaches to prepare the coatings of these ceramics. This work investigates the possibility of applying an aqueous sol-gel method to synthesize pure phases of YIG, YIP, and TIP, then adapting the method to fabricate ceramic YIG, YIP, and TIP coatings using a dip-coating process (which is a process that finds use in an industrial setting as well).

## 2. Materials and Methods

Yttrium nitrate (Y(NO_3_)_3_∙6H_2_O, Alfa Aesar, Haverill, MA, USA, 99.9%) was used as purchased, while terbium nitrate was made from terbium oxide (Tb_4_O_7_, Alfa Aesar, 99.9%). For this purpose, 0.8124 g (1.0865 mmol) of terbium oxide (Tb_4_O_7_) was added to 8 mL of concentrated nitric acid (HNO_3_, Eurochemicals, Quezon, Philippines, 65%). Nitric acid was then evaporated using a heated magnetic stirrer (without letting the solution reach the boiling point). For a more complete removal of nitric acid, a small amount of deionized water was added, then evaporated, repeating the cycle three times. The remaining solid terbium nitrate was used as-prepared in the sol-gel synthesis that followed.

Previously reported sol-gel synthesis processing was adapted as needed for the preparation of YIG, YIP, and TIP [27,28,29]. For the sol-gel synthesis, the nitrates were dissolved in 20 mL of deionized water (3.1142 g (8.1309 mmol) yttrium nitrate for yttrium iron garnet (YIG); 4.7653 g (12.442 mmol) for yttrium iron perovskite (YIP); as-synthesized terbium nitrate for terbium iron perovskite (TIP)). Then, a stoichiometric amount of iron nitrate (Fe(NO_3_)_3_·9H_2_O, Duro-Galvanit-Chemie, Herne, Germany, 98%) was dissolved in 50 mL (or 25 mL in case of terbium) of deionized water. The solutions were mixed using a magnetic stirrer in covered beakers for 1 h at 60 °C. After that, ethylene glycol ((CH_2_OH)_2_, Aldrich, St. Louis, MO, USA, 99.5%) was added (in a 1:1 molar ratio to metal ions in the solution) as a complexing agent and left to stir for an additional 2 h. Part of the solution (10 mL) was then separated for gelation and sintering to obtain powders, the rest was kept for the fabrication of coatings. To obtain ceramic powders of the materials, the separated solution was placed in an uncovered beaker and stirred at 60 °C until the gel formed. After the formation of the gel, the temperature was raised to 120 °C and the gel was left to dry for 24 h. The obtained material was removed from the beaker, ground into a fine powder and heated for 2 h at 800 °C in a furnace using a heating rate of 10 °C/min. The obtained powders were then sintered again at 1000 °C for 10 h using a heating rate of 1 °C/min.

Synthesis of powders was followed by adapting the sol-gel procedure for the fabrication of coatings. Thin films of YIG, YIP, and TIP were deposited on a p-type silicon substrate (CrysTech, Qingdao, China) using a dip-coating technique. First, to prepare the silicon substrates, 2 × 1 cm sized p-type silicon (surface plane 100) substrates were placed in a freshly prepared 3:1 mixture of H_2_SO_4_ and H_2_O_2_ (commonly known as the “piranha solution”) and left there for 15 min. The substrates were then removed, washed using running deionized water, and left in a dilute 2% solution of hydrofluoric acid (HF) for 5 min. Again, the substrates were then removed and washed under running deionized water. The surface was then activated using oxygen plasma. The previously mentioned solutions of metal salts and ethylene glycol were mixed with a 3% solution of polyvinyl alcohol ((C_4_H_6_O_2_*C_2_H_4_O)n, partially hydrolyzed, Mw approx. 70,000, Merck Schuchardt OHG, Hohenbrunn, Germany) at a ratio of 1:1. Dip coating procedure was then carried out, using the prepared silicon substrates and the mentioned solution, with a submerging speed of 85 mm/min, a retention time of 10 s, and a retrieval speed of 40 mm/min. After the dip-coating procedure, the newly formed coatings were left to dry for 30 min, then placed in a heating oven and heated at a rate of 1 °C/min up to 500 °C, with a retention time of 1 h. This coating-heating cycle was repeated 5 or 15 times, after which the final heating was up to 1000 °C for 10 h, so the ceramic would form [28].

Simultaneous thermal analyzer Perkin Elmer STA 6000 (Waltham, MA, USA) (gels were heated in an airflow (20 mL/min) at a heating rate of 10 °C/min) was used for thermogravimetry/differential scanning calorimetry (TG/DSC) measurements of the precursor gels. X-ray diffractometer Rigaku MiniFlex II (The Woodlands, TX, USA) operating with Cu K_α1_ radiation under Bragg-Brentano configuration was used for the characterization of synthesized materials. Scanning electron microscope FE-SEM Hitachi SU 70 (Tokyo, Japan) was used to investigate the surfaces and cross-section of the coatings. Veeco Bioscope 2 atomic force microscope (Les Ulis, France) was used to determine the surface roughness of the coatings. Mössbauer spectrometer (Wissenschaftliche Elektronik GmbH, Starnberg, Germany; ^57^Co(Rh) source) was also employed, as well as a magnetometer consisting of a lock-in amplifier SR510 (Stanford Research Systems, Sunnyvale, CA, USA), a gauss/teslameter FH-54 (Magnet Physics), and a laboratory magnet supplied by the power source SM 330-AR-22 (Delta Elektronika, Zierikzee, The Netherlands) (magnetic and Mössbauer measurements carried out in Centre for Physical Sciences and Technology). The annealing of materials was performed in an ordinary furnace (SNOL E5CN-H, Utena, Lithuania). For the dip-coating process, a KSV Dip Coater D equipment (Espoo, Finland) was used. For the activation of substrate surface the plasma cleaning with an equipment Nano, Diener Electronic (Ebhausen/Germany) was used.

## 3. Results and Discussion

**Figure 1:**
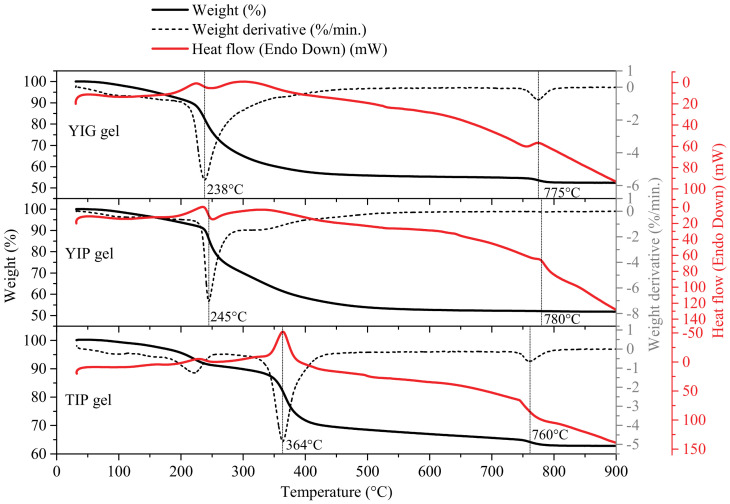
TG/DTG/DSC curves of indicated gels.

Thermogravimetric analysis was performed for the gel precursors that were later used to obtain YIG, YIP, and TIP ceramics. The thermogravimetry/differential thermogravimetry/differential scanning calorimetry TG/DTG/DSC results (Figure 1) show that there are three main thermal processes visible for all compounds. The first mass loss occurring until about 200 °C could be attributed to the removal of moisture and water from hydrates. The second event is a major loss of mass at either around 250 °C (for yttrium compounds) or 360 °C (for the terbium compound). This mass loss corresponds to the decomposition of organic parts of the gels and the nitrates [30]. The DSC curve shows exothermic peaks in that temperature range supporting the TG results obtained. Also, the fact that the main mass loss peak that corresponds to gel decomposition is located between the decomposition temperatures for a pure iron or pure yttrium gels of this type (as opposed to having two separate peaks at these points) indicates that most likely a mixed-metal gel was formed, rather than two separate gels for each oxide [30,31,32]. The third thermal event at around 780 °C most likely corresponds to the final formation of the crystal phase. The mass loss probably corresponds to the decomposition of the intermediate oxycarbonate Y_2_O_2_CO_3_ to the oxide which occurs in the temperature range 600–800 °C depending on the atmosphere used [33]. The small, mostly exothermic heat flow peak, meanwhile, probably also indicates the decomposition of the intermediate oxycarbonate as well as the crystal phase formation itself. This has been reported in other, similar sol-gel processes for YIG before [34]. This, however, does not necessarily mean that this temperature is sufficient to obtain pure phases, as it has been reported that a pure garnet phase may not form until about 1000 °C. A previous report suggests that initially, perovskite is a major phase that forms, with the amount of garnet increasing with increased temperature [27]. The formation of perovskites occurs at around the same 760–780 °C, followed only by an increase of crystallinity with further increasing temperature. The primary reason for thermogravimetric analysis in this particular case was to determine the temperature range suitable for experimentation for this particular system and what processes can be expected.

**Figure 2:**
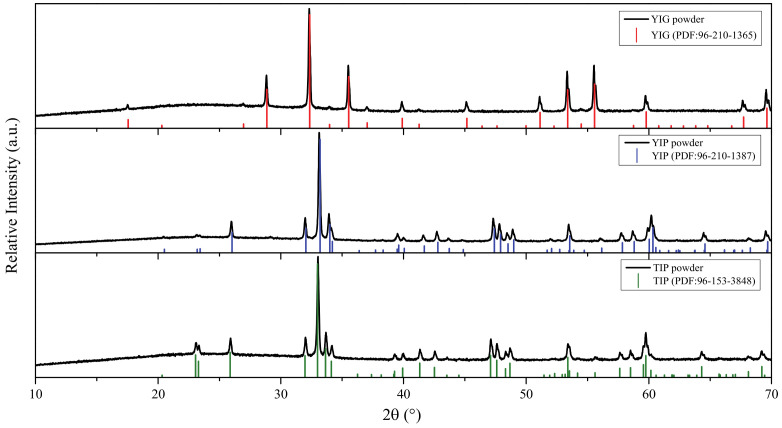
XRD patterns of yttrium iron garnet (YIG), yttrium iron perovskite (YIP), and terbium iron perovskite (TIP) powders obtained by the aqueous sol-gel method.

The XRD profile matching results (Figure 2) confirm that after heating the gel precursors, monophasic YIG, YIP, and TIP powders formed. The XRD patterns show well-defined diffraction peaks that match the standard patterns very well. Thus, the synthesis procedure chosen is suitable for obtaining pure crystalline phases. This is useful because it shows that the same sol-gel process can be used not only for the synthesis of YIG, as was described by other authors [27,28], but can also be adapted to obtain pure YIP and TIP, and potentially many other similar structures in a very simple and inexpensive way. Confirming that the simple sol-gel method was successful in obtaining powders allowed to expect positive results for the production of coatings as well.

**Figure 3:**
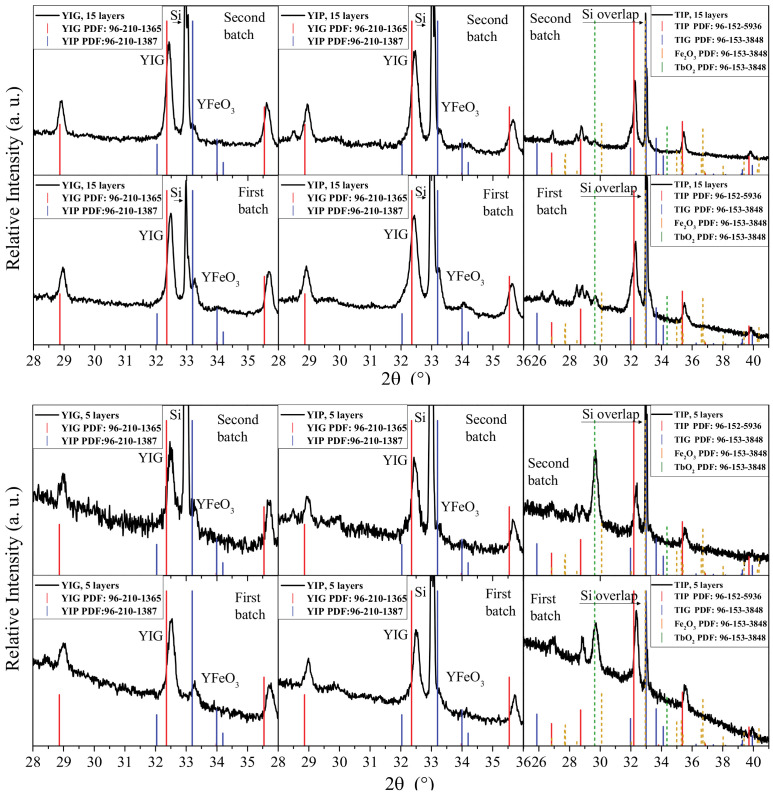
XRD patterns of 5-layer (**bottom** patterns) and 15-layer (**top** patterns) YIG, YIP, and TIP coatings obtained by sol-gel method using a dip-coating procedure. “First batch” indicates the initial experiment, while “Second batch” indicates the repeated experiment.

With this in mind, the experiments then moved onto the task of producing coatings. A dip-coating process was chosen for this, again, because it is simple to upscale, inexpensive, and versatile, allowing objects of various shapes and sizes to be coated. As already mentioned, the coatings play a very important role in the applicability of similar ceramics. The XRD patterns of the coatings obtained on silicon substrates are presented in Figure 3. The “first batch” and “second batch” labels are referencing the fact that the experiment was carried out a second time, to ensure the results showed repeatability. Only the area containing the defining peaks is presented for all samples for the sake of clarity. Several features are immediately notable. First, there is an intense peak caused by Si monocrystal visible for all samples. This presents several problems. One of them is that the main peak of iron oxide coincides with it, complicating the process of determining whether any remnant iron oxide is present. The second problem is that it partially overlaps with the main peak of YFeO_3_, making it more difficult to identify. It also near-perfectly overlaps with the main peak of TbFeO_3_, which means that for terbium samples, the presence of terbium perovskite cannot be confirmed or rejected definitely—only assumptions can be made based on the fact that Si peak is very narrow, while the sudden widening of the peak at the bottom might indicate the overlapping of the TbFeO_3_ peak. The diffraction patterns still provide useful data, as long as these complications are kept in mind.

It is interesting to note that in all cases a mixture of both garnet and perovskite phases formed, regardless of the nominal stoichiometry of metals. Since the same procedures worked well for obtaining bulk pure phases, this difference is most likely caused by the surface interaction, since that is the most apparent and impactful change that occurred when compared to powder synthesis. The resulting XRD patterns of coatings are nearly identical in the ratio between the main peaks of the two Y_3_Fe_5_O_12_ and YFeO_3_ phases in each coating. It is possible that the stoichiometry of the gel that ends up on the silicon substrate is different from the stoichiometry of the solution itself. Otherwise, if the gel stoichiometry of the gel deposited on the silicon substrate corresponded to YIG or YIP, one could expect the formed secondary phases to be different. The conclusion that can be drawn from these results is that to obtain phases of higher purity, the coating process itself would need to be optimized. The fact that pure powders could be obtained in the first experiments also presents a compelling argument supporting the idea that if the coating process was adapted to minimize the surface influence in the gel retention step perhaps pure coatings could also be obtained.

**Figure 4:**
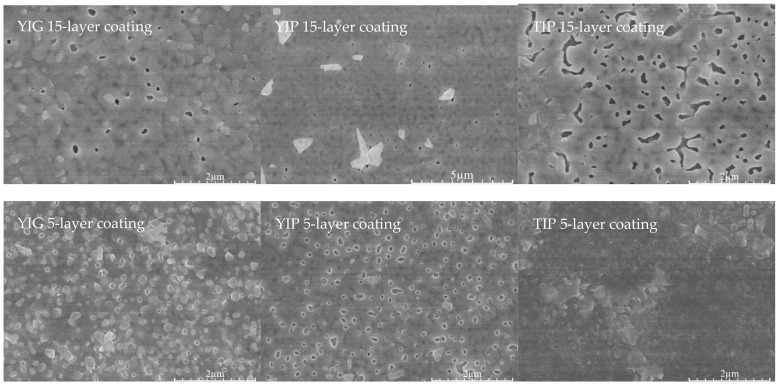
SEM micrographs of the surfaces of sol-gel derived YIG, YIP, and TIP samples.

SEM micrographs (Figure 4) revealed that the surface of the coatings was composed of different crystalline particles with slightly different degrees of porosity (a relatively common occurrence for the dip-coating process [35]).

**Figure 5:**
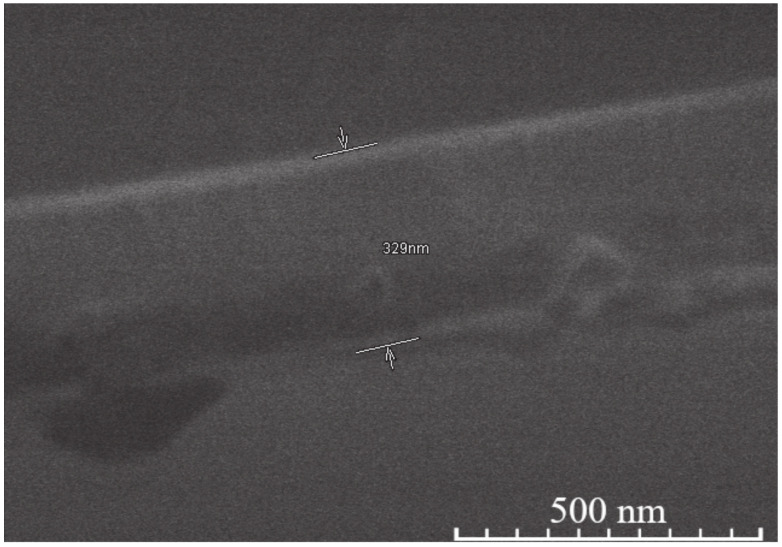
SEM micrograph of a cross-section of one 5-layer YIP sample, with example of thickness measurement.

On the other hand, the microstructure of the sol-gel-derived YIG, YIP, and TIP coatings is more similar than different, i.e., it is almost identical for all compositions. Cross-section SEM images of the coatings were also analyzed. The representative SEM micrograph is presented in Figure 5. This provided the opportunity to measure the thickness of the coatings. These measurements are important for several reasons. The thickness of the coating that can be obtained is often a major factor in the application of the material. Because of this, it is important to know what range of thickness can be expected. The obtained results are summarized in Table 1. Coating thickness for the samples with 5-layer varies in the range of 230–310 nm, while the 15-layer samples had an average thickness in the range of 380–520 nm. It can be noted that a higher number of layers decreases porosity, and possibly increases crystallinity.

**Figure 6:**
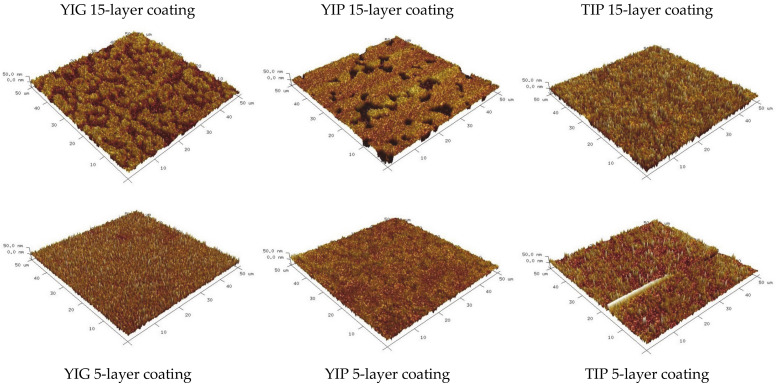
Atomic force microscopy results for indicated coatings.

The surface morphology of the coatings was also characterized by AFM. The characteristic images of the coatings observed in this study are presented in Figure 6. The average roughness parameters are given in Table 2. As can be seen, the average roughness values are within the range of 6–11 nm, which is reasonable for this type of synthesis process. The 15-layer samples show a slightly higher roughness than the 5-layer ones. The most notable feature was that the 15-layer coatings seemed to have more expressed indentations or pores, whereas the 5-layer coatings were more uniformly uneven.

**Figure 7:**
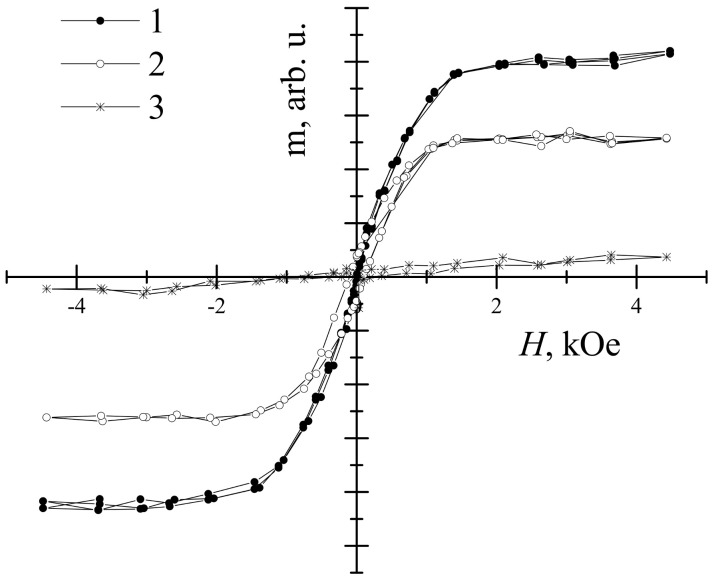
Magnetization hysteresis of: 1—YIG, 2—YIP, and 3—TIP thin layers. Magnetic field is perpendicular to the layer surface.

**Figure 8:**
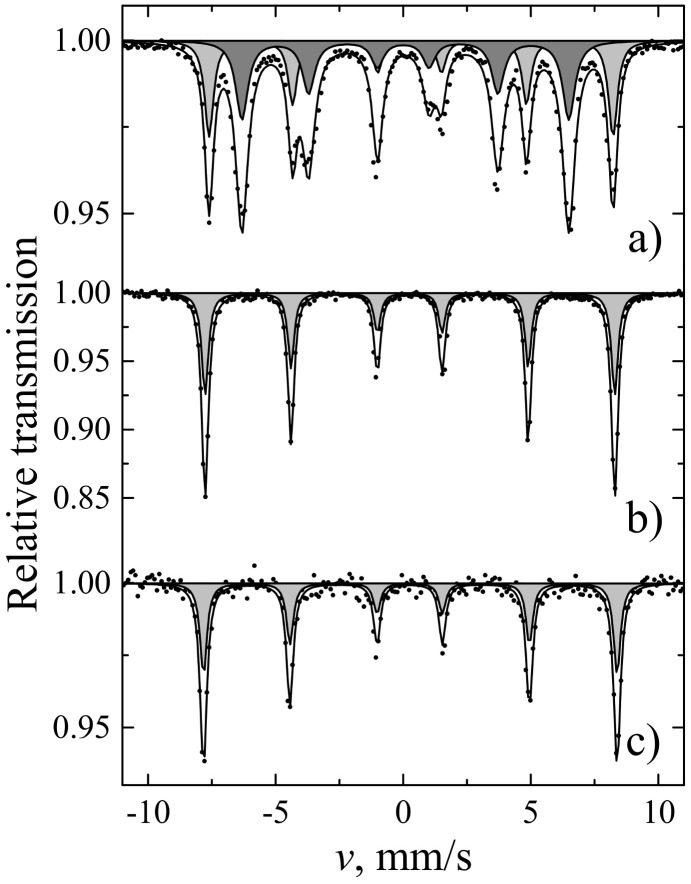
Mössbauer spectra for YIG (**a**), YIP (**b**), and TIP (**c**) bulk samples at room temperature.

**Figure 9:**
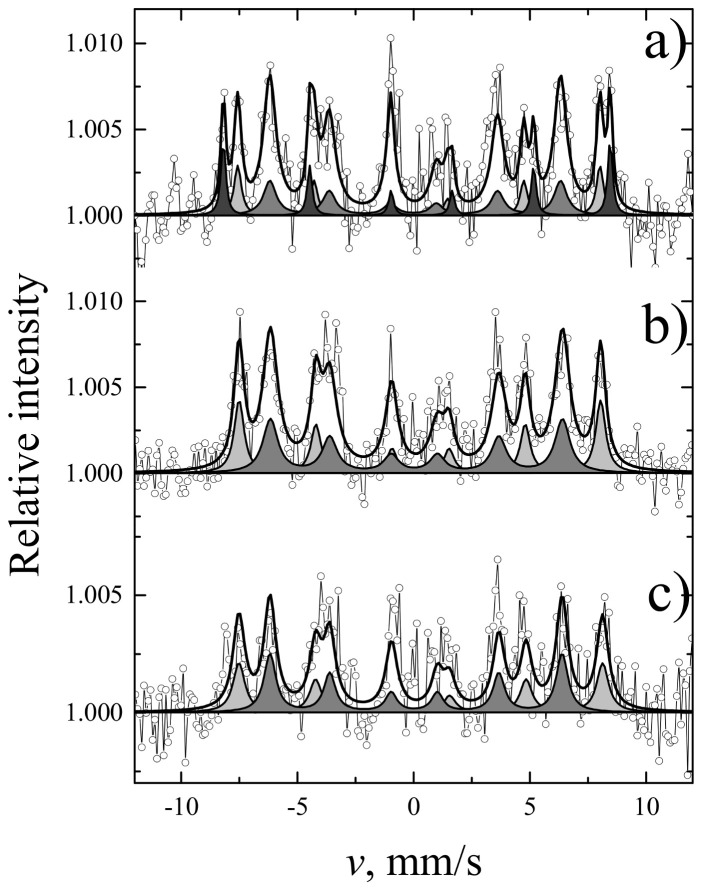
Conversion electron Mössbauer spectra of (**a**) YIG, (**b**) YIP, and (**c**) TIP layers.

Finally, the magnetic measurements were performed for the 2D samples (Figure 7). The results of Mössbauer spectroscopy obtained for the coatings were compared with those determined for bulk materials (Figure 8 and Figure 9, and Table 3 and Table 4). Magnetization dependencies show saturation magnetization that is characteristic of the ferrimagnetic materials. The saturation magnetization of TIP is several times lower than that of YIG and YIP. For all coating samples, Mössbauer subspectra of a and d sublattices (Table 4) of iron garnets were observed. However, their area ratios for YIG did not match the theoretical expectations well, as such, α-Fe_2_O_3_ was also not excluded. Even so, the most intense d sublattice subspectrum indicates that garnet is the dominant phase.

## 4. Conclusions

The simple aqueous sol-gel method using inorganic salts as starting materials was found to be suitable for the preparation of monophasic yttrium iron garnet (YIG), yttrium iron perovskite (YIP), and terbium iron perovskite (TIP) three-dimensional powders. The same methodological approach was transferred for the fabrication of YIG, YIP, and TIP thin films. The resulting XRD patterns of the coatings are nearly identical in the ratio between the main peaks of the two Y_3_Fe_5_O_12_ and YFeO_3_ phases in each coating. It was concluded from the XRD data that in all cases, regardless of the nominal stoichiometry, a mixture of both garnet and perovskite two-dimensional phases formed in a reproducible manner. It is possible that the stoichiometry of the gel that ends up on the silicon substrate is different from the stoichiometry of the solution itself. The conclusion that can be drawn from these results is that to obtain phases of higher purity, the coating process itself would need to be optimized. SEM micrographs and AFM images revealed that the microstructure of sol-gel-derived YIG, YIP, and TIP coatings is almost identical for all compositions. Coating thickness varied from sample to sample, with 5-layer samples having an average thickness in the range of 230–310 nm, whereas the 15-layer samples had an average thickness in the range of 380–520 nm. The average roughness values were found to be within the range of 6–11 nm. The 15-layer samples, however, showed a slightly higher roughness than the 5-layer ones. Magnetization dependencies show saturation magnetization that is characteristic of ferrimagnetic materials. The saturation magnetization of TIP is several times lower than that of YIG and YIP. Analysis of Mössbauer subspectra revealed garnet phase to be the major one in the 2D samples.

## Figures and Tables

**Table 1:** Thickness of YIG, YIP, and TIP 15- and 5-layer coatings.

Sample		Thickness Range, Nm	Average Thickness, Nm	Thickness Median, Nm	Short Analysis *
15-layer	YIG	380–472	424	416	Mostly even surface. No noticeable pores or cavities. Difficult to measure since separation line between coating and Si not clearly visible.
	YIP	485–559	511	506	Moderately uneven surface. Few pores, some cavities visible.
	TIP	358–414	386	385	Mostly even surface, however either cavities or pores clearly visible.
5-layer	YIG	201–342	273	280	Uneven surface, some unusually large or small crystallites on the surface expand thickness range. No hidden cavities visible.
	YIP	261–354	307	316	Even surface. Few cavities or pores visible.
	TIP	216–256	233	234	Even surface, thickness variations due to nature of dip coating. No hidden cavities visible.

* Note that the cavities/pores may have potentially formed during the snapping process rather than just synthesis, so should be taken into account in conjunction with regular SEM images.

**Table 2:** Average (R_a_) and root mean square (R_q_) roughness of indicated dip-coating samples as measured by AFM.

	R_q_, nm	R_a_, nm
YIG 15-layer sample	10.7	8.7
YIG 5-layer sample	10.1	7.9
YIP 15-layer sample	12.5	8.8
YIP 5-layer sample	8.56	6.5
TIP 15-layer sample	13.6	10.7
TIP 5-layer sample	10.6	7.1

**Table 3:** Data from bulk sample Mössbauer spectra measured at room temperature: S is the relative area of subspectrum, Γ, δ, Δ(2ε), and B are line width, isomer shift, quadrupole shift, and hyperfine field, respectively.

	Sites	S, %	Γ, mm/s	δ, mm/s	2ε, mm/s	B, T
YIG	a	39	0.42 ± 0.01	0.38 ± 0.01	0.06 ± 0.01	49.07 ± 0.01
	d	61	0.58 ± 0.01	0.15 ± 0.01	0.08 ± 0.01	39.71 ± 0.01
YIP	-	100	0.31 ± 0.01	0.36 ± 0.01	0.01 ± 0.01	49.78 ± 0.01
TIP	-	100	0.32 ± 0.01	0.37 ± 0.01	0.02 ± 0.01	50.25 ± 0.02

**Table 4:** Data from conversion electron Mössbauer spectra measured at room temperature for YIG, YFO and TFO layers on Si: S is relative area of subspectrum, Γ, δ, 2ε, and B are line width, isomer shift, quadrupole shift, and hyperfine field, respectively.

	S, %	Γ, mm/s	δ, mm/s	2ε, mm/s	B, T	
YIG	26 ± 3	0.37 ± 0.06	0.33 ± 0.02	0.00 ± 0.03	48.29 ± 0.15	IG a sites
	56 ± 3	0.66 ± 0.06	0.13 ± 0.02	0.08 ± 0.04	38.76 ± 0.13	IG d sites
	17 ± 3	0.25 ± 0.05	0.35 ± 0.01	−0.21 ± 0.02	51.58 ± 0.09	α-Fe_2_O_3_
YIP	37 ± 2	0.51 ± 0.05	0.39 ± 0.02	−0.03 ± 0.03	48.20 ± 0.11	IG a sites
	63 ± 2	0.77 ± 0.06	0.18 ± 0.02	0.09 ± 0.03	38.94 ± 0.12	IG d sites
TIP	46 ± 5	0.62 ± 0.10	0.42 ± 0.03	−0.00 ± 0.06	48.49 ± 0.22	IG a sites
	54 ± 5	0.61 ± 0.08	0.17 ± 0.03	0.08 ± 0.05	38.95 ± 0.18	IG d sites

## Data Availability

Data is contained within the article.
